# Cognitive memory screening and referral program in community pharmacies in the United States

**DOI:** 10.1007/s11096-013-9904-7

**Published:** 2013-12-20

**Authors:** Nathaniel M. Rickles, Jann B. Skelton, Jennifer Davis, Jennifer Hopson

**Affiliations:** 1 Northeastern University, Boston, MA USA; 2 Silver Pennies Consulting, Inc, North Caldwell, NJ USA; 3 Fred Meyer Pharmacy, Brookings, OR USA; 4 Continuing Education Department, Oregon State University College of Pharmacy, Corvallis, OR USA; 5 Kerr Drug, Lenoir, NC USA

**Keywords:** Alzheimer’s disease, Ambulatory care, Clinical pharmacy services, Cognitive memory screening, Community pharmacy, Dementia, Physician referral

## Abstract

*Introduction* 12 chain community pharmacy sites located in two geographic areas with the United States implemented easy-to-administer memory screening assessments for patients with risk factors of cognitive memory decline and referred at-risk patients to their physicians. *Aim of the study* To evaluate the impact of a pharmacy-based cognitive memory screening and referral program, measure patient satisfaction with these advanced clinical services, and assess willingness to pay for cognitive memory screening services. *Setting* 12 chain pharmacy sites located in two geographic areas—ten Fred Meyer Pharmacies located in the Portland, Oregon area and two Kerr Drug Pharmacies located in North Carolina. *Method* Pharmacists were educated on Alzheimer’s disease, trained on how to provide cognitive memory screening exams, and equipped with screening and documentation tools. Following each screening, pharmacist provided education and counseling to the patients and referred at-risk patients to physicians for follow-up as appropriate. *Main outcome measures* Results of screenings; satisfaction of patients; willingness to pay. *Results* Pharmacists delivered cognitive memory assessments to 161 patients from June to November 2008. 44.1 % of patients experienced at least one cognitive deficiency that required referral to a physician based on the screening conducted. The cognitive memory screening and referral program was highly regarded by patients who completed the satisfaction survey, with 98.4 % of respondents indicating that they were either very satisfied or satisfied with the program. *Conclusion* Cognitive memory screening can be easily incorporated into clinical service offerings in community pharmacy practice and provides a valuable opportunity to identify patients at-risk and refer them to a physician for appropriate testing and diagnosis.

## Impact of findings on practice

- Cognitive memory screening can be easily incorporated into clinical service offerings in American community pharmacy practice.

## Introduction

Alzheimer’s disease (AD) is a serious, progressive and fatal type of dementia that destroys brain cells and causes problems with memory, thinking, and behavior. AD is the most common type of dementia and is the sixth-leading cause of death in the United States [1]. Almost two-thirds of Americans with AD are women. There is no cure for patients diagnosed with dementia, and its prevalence increases dramatically with age. An estimated 36 million people currently have dementia worldwide [2], including over 5 million Americans with AD [3]. Over 115 million people across the globe will have dementia by 2050 [2], and in America, the number of people aged 65 years or older with AD could increase by 50 % by 2050 [4].

Additionally, the over $600 billion international economic impact of dementia is staggering [2]. The annual cost to care for an American individual with dementia is $56,290 [5]. The direct and indirect financial costs to care for people in the United States with AD amount to more than $200 billion annually, and the economic value of the care provided by family and other paid caregivers of people with AD and other dementias is an additional $210 billion [4]. People with AD in America have more than three times as many hospitals stays as other older people and their total Medicare costs were nineteen times higher than for other Medicare beneficiaries without AD and other dementias [4].

Diagnosis of AD is complicated and can involve a combination of detailed medical history, physical examination, laboratory testing, cognitive assessments, and brain-imaging scans conducted by a physician [3]. A definitive diagnosis of AD can only be determined with an examination of the brain upon autopsy. There are no treatment options to stop the deterioration of brain cells in AD. However, there are benefits of early detection, including [4]:

- early initiation of drug treatment to treat and delay the worsening of symptoms;
- ability to engage in planning for future financial and healthcare needs;
- engagement in support groups; and
- ability to make lifestyle changes before the disease progresses further.

As highly accessible health care providers, pharmacists are in an ideal position to identify and assist in the management of individuals with AD and other cognitive memory disorders. The cognitive memory screening and referral program (CMSRP) is the first project to measure the impact of a pharmacist-based cognitive memory screening service delivered in community pharmacy practice in the United States. Although community pharmacy workflow processes may differ between the US and other countries, this program was designed to be similar to previously implemented chronic disease screening events, making it replicable in any setting where these types of screening events have occurred. While the involvement of community pharmacists in the screening of AD is novel in the US and across the globe, there are several studies supporting community pharmacists having a significant beneficial role in screening of diseases. There have been several recent examples of enhanced care through community pharmacy involvement in screening efforts both in and outside of the US [6–9].

Utilizing a combination of self-assessment surveys, blood tests and other biological measurements, and physical assessments, pharmacist-provided clinical screening services for diseases such as cardiovascular disease [10], osteoporosis [11], depression [12], and diabetes [13] among others have successfully improved identification of patients at-risk, provided disease state and medication education, and improved clinical outcomes. Some of these interventions have resulted in lifestyle changes in those patients who were identified as at-risk, highlighting the potential of community pharmacists to improve the quality of life of the general population [14].

Pharmacists also have a unique opportunity to identify and assist in the management of individuals with AD. A growing body of evidence suggests that the health of the brain is closely linked to the overall health of the heart and blood vessels. Some data indicate that management of modifiable cardiovascular risk factors, such as high cholesterol, Type 2 diabetes, high blood pressure, smoking, obesity and physical inactivity may help avoid or delay cognitive decline [15–23]. The strong link between brain health and heart heath provides a unique opportunity for pharmacists to expand their clinical services to provide care to patients at-risk of developing AD and to provide additional services to patients that are already being managed by their pharmacist for other conditions.

Health education and disease awareness activities consisted of a series of memory screening assessments, patient and caregiver education, and physician referrals. The project also included other wellness and support services in an effort to address the spectrum of patient, caregiver and provider needs.

## Aim of the study

This manuscript describes the initial patient screening activities, follow-up, and results of patient referrals. The primary objective of the CMSRP was to evaluate the impact of pharmacy-based cognitive memory screening activities with a focus on early detection of AD, appropriateness of referral of at-risk patients to their primary care physicians for potential diagnosis, and outcomes of physician referral related to follow-up. The program also measured patient satisfaction with advanced clinical services and the willingness of patients to pay for cognitive memory screening services.

## Method

The CMSRP was initiated in June of 2008 in conjunction with 12 chain pharmacy sites located in two geographic areas (10 community pharmacies located in the Oregon and two community pharmacies located in North Carolina). Within a 6-month timeframe, the participating pharmacies screened a total 161 patients that were identified as at-risk of developing AD.

### Patient description

Services were offered to patients identified within the community pharmacy and through awareness activities such as posters and informational brochures, which included information about the warning signs of AD and the availability of the screenings. Pharmacists delivered screening services to patients by appointment, through stand-alone screening days and through outreach to local assisted living facilities and senior centers.

Patients were offered screening services by a pharmacist if they self-identified as having at least one warning sign for AD as outlined by the Alzheimer’s Association or if the pharmacist assessed that the patient could benefit from the service based on factors such as age, co-morbid health conditions and/or observation of behaviors. Warning signs for AD are listed in Table 1.

**Table 1:** Warning signs for Alzheimer’s disease [24]

Warning signs for Alzheimer’s disease
Memory loss that disrupts life
Challenges in planning and problem solving
Difficulty performing familiar tasks at home, work or at leisure
Confusion with time or place
Trouble understanding visual and spatial relationships
New problems with words in speaking or writing
Misplacing things and losing the ability to retrace steps
Decreased or poor judgment
Withdrawal from work or social activities
Changes in mood or personality

As part of the screening process, all patients and caregivers were provided with educational information to promote understanding of AD and maintaining brain health. Patients were required to complete a consent form and a health risk assessment (HRA) prior to obtaining a cognitive memory screening from the pharmacist.

### Pharmacist training

The participating pharmacists from each study site were trained via a 2-hour live Webinar training program developed and delivered by the APhA Foundation that provided:

- An overview of the American Pharmacists Association (APhA) Foundation CMSRP Program;
- A clinical update on AD;
- A review of the patient care process;
- A detailed overview of study forms and paperwork;
- Strategies for patient identification;
- Training on the use of memory screening instruments; and
- A protocol review for patient follow-up, data collecting and reporting.

### Screening tools

The memory screening tools were selected based on the ability to provide meaningful results to patients and physicians, the ease of implementation in a community pharmacy setting, and the small time commitment for both the pharmacist and the patient. These tools can also be used in conjunction with other clinical services or educational programs that the pharmacy may already offer (i.e., Medication Therapy Management (MTM) services, blood pressure monitoring, or diabetes screening).

The validated instruments used for patient screening included the Three-Word Recall [25], the Clock Draw Test [26], and the Animal Fluency Test [27]. The combination of the Three-Word Recall and the Clock Draw Test is also called the Mini-cog [28]. The Three-Word Recall tests a patient’s ability to recall and retain information, both elements of abstract thinking. Difficulty in abstract thinking is a component of AD. The Clock-Draw Test assesses a patient’s ability to retain and recall pre-existing relevant information. The scores of the Clock Draw Test are used in conjunction with the scores of the Three Word Recall to determine the most appropriate referral recommendation for the patient.

The Animal Fluency Test is a categorical test or word fluency test, which is a common and reliable type of word recall test used to assess patients at-risk for AD or other cognitive memory disorders [27]. Word recall tests measure short-term forgetfulness and impairment in word-finding capability, verbal production, noun-retrieval, semantic memory, and language.

### Patient counseling

Following the administration of cognitive memory screening assessments during screening events, pharmacists provided counseling and disease education to patients and caregivers as required. Counseling was customized based on patient characteristics, medical history, medication therapy and responses to the HRA questions, and length of counseling sessions varied based on the individual patient’s needs. Counseling elements included a discussion about the difference between screening activities and physician diagnosis, the common reasons for cognitive memory decline (i.e., sleep disturbances, depression, stress, or other medications), and options for potential follow-up. If the patient required referral to the physician, pharmacists explained that it was for follow-up and evaluation. They also provided the patient with a copy of the follow-up fax to the physician if warranted.

### Risk stratification and physician referral

Assessment scoring guidelines were used by pharmacists to refer patients for physician follow-up. Mini-cog indications for referral were used, which recommends referral for all patients with a Clock Draw Test score of 0–3 who also have a three-Word Recall Test score of 0–3. Patients with a Clock Draw Test score of four and a three-Word Recall Test score of 0 should also be referred [28]. Additionally if assessment scores demonstrated a cognitive deficiency in any of the three tests, patients could be referred to the physician. The pharmacist used their professional judgment based on the patient assessment form, medical history, screening results and patient interaction in their decision to refer a patient for physician follow-up. The scores for each patient assessment and the resulting action taken by the pharmacist were documented. Patients received a verbal referral from the pharmacist and, in cases of severe cognitive deficiency, the pharmacist directly contacted the patient’s physician by phone or fax to report screening results.

### Main outcome measures

#### Results of screening

Results of screening was evaluated by identifying how many patients pharmacists appropriately referred and did not refer to their physicians for further evaluation, how many patients referred planned to go to their physicians for follow-up, and how many of those referred patients actually followed up with their physicians. The present evaluation did not explore other outcomes of referral such as earlier initiation of treatment, ability to plan future financial and healthcare needs, engage in support groups, and/or ability to make lifestyle changes in advance of disease progression.

#### Patient satisfaction and willingness to pay

Patient satisfaction with services was evaluated through two mechanisms: the completion of a voluntary participant satisfaction survey and through follow-up phone calls from the pharmacist to participating patients 45–90 days after the initial pharmacy-based screening. This time frame allowed sufficient time for patients to follow up with their physician. Patient satisfaction surveys were provided to patients at the time they received their screening assessment. A stamped and addressed envelope was provided to return the surveys to the APhA Foundation. Patient satisfaction questions were measured on a five-point Likert scale ranging from very satisfied to very dissatisfied. Follow-up phone-interviews were conducted with patients who were referred for follow-up with a physician to determine the outcomes of physician follow-up (i.e., if they were prescribed medications or had additional testing) and willingness to pay for memory screening services.

## Results

The participating pharmacies identified and screened 161 patients with more than one warning sign for AD. Social and clinical demographics in Table 2 show that of these patients, 118 (73.8 %) were female, 124 (77.1 %) had a high school or college education, and 146 (91.8 %) were living at home. The mean age of participants was 65 years. Of the 112 patients (69.4 %) that had no family history of AD, 66 (58.9 %) had risk factors for memory loss and 46 of those patients (69.6 %) were not being treated for memory loss. Table 3 displays that among patients who were identified as having warning signs of AD, the most frequent signs reported include trouble remembering names (n = 85, 52.8 %), needing reminders to do things (n = 78, 48.4 %), and misplacing car keys and other items (n = 60, 37.3 %).

**Table 2:** Social and clinical demographics of participating patients (n = 161)

Demographic:	n (%)
Highest level of education	
High school	69 (42.9)
College	55 (34.2)
Graduate school	19 (11.8)
Current living situation	
Home	146 (91.8)
Assisted living	8 (5.0
Other	5 (3.1)
Family history of AD	49 (30.6)
Previous diagnosis of dementia	3 (1.9)
Previous diagnosis of stroke	16 (10)
Previous diagnosis of head injury	34 (21.4)
Risk factors of memory loss	93 (58.9)
Treated for memory loss	48 (30.4)

**Table 3:** Warning signs of potential memory loss (n = 161)

Warning sign	n (%)
Trouble remembering names	85 (52.8)
Need reminders to do things	78 (48.4)
Misplaces car keys and other items	60 (37.3)
Forgets appointments	51 (31.7)
Repeats conversations	41 (25.5)
Family member with Alzheimer’s disease	38 (23.6)
Lost interest in hobbies and social events	35 (21.7)
Gets angry easily	32 (19.9)
Trouble finishing a sentence	30 (18.6)
Asks same questions repeatedly	29 (18.0)
Trouble reading books	23 (14.3)
Gets lost easily	18 (11.2)
Loss of smell	17 (10.6)
Has trouble making change for a purchase	6 (3.7)
Needs help eating and dressing	3 (1.9)

Based on the Alzheimer’s testing scores listed in Table 4, pharmacists identified 71 patients (44.1 %) with at least one cognitive deficiency that required referral to a physician based on the three screening assessments conducted. Pharmacists used their professional judgment when referring patients to their physician, referring 54 screened patients (33.5 %) to assess the cause of cognitive memory decline. Eight additional patients, who did not show cognitive deficiencies in the screening exercises, were also referred based on the pharmacist’s clinical judgment. Overall, 118 patients (73.2 %) received an appropriate recommendation by a pharmacist. An appropriate referral recommendation included (1) patients did not qualify for referral and therefore no physician referral was made, or (2) referral to a physician was indicated and was made. After receiving the pharmacist’s initial intervention, 23 referred patients (69.7 %) indicated that they planned to go to the physician for follow-up).

**Table 4:** Alzheimer’s testing and referral results (n = 161)

Item	n (%)
Word recall score	
0	6 (3.7)
1	17 (10.6)
2	39 (24.2)
3	99 (61.5)
Animal fluency score	
<15 animals listed	59 (36.6)
≥15 animals listed	102 (63.4)
Clock drawing score	
0	1 (0.6)
1	1 (0.6)
2	8 (5.0)
3	24 (14.9)
4	127 (78.9)
Need for referral based on three-word recall, clock draw and/or animal fluency	
Referral needed	71 (44.1)
No referral needed	90 (55.9)
Pharmacist-reported refer to MD	
Yes	54 (33.5)
No	91 (56.5)
Not recorded	16 (9.9)
Extent of pharmacist referral based on need	
No indication of referral, RPh referred anyway	8 (5.0)
Test indicated referral was needed, RPh did not make referral	19 (11.8)
Referral was indicated and was made	46 (28.5)
Did not qualify for referral and no referral was made	72 (44.7)
Referral status not recorded	16 (9.9)

Shown in Table 5, 39 patients (72.2 %) who were referred to the physician completed the follow-up phone survey with the pharmacist. Of those telephone survey respondents, 22 patients (56.4 %) were willing to pay out-of-pocket for screening services. Seventeen patients (77.2 %) who were willing to pay indicated that they would be willing to pay five to ten dollars for pharmacist services. Interestingly, there was an association with willingness to pay and a screening result that indicated some level of cognitive memory decline. This exemplifies that high-risk patients are concerned with their health, recognize the importance of screening for health problems, and are willing to compensate pharmacists for more accessible services.

**Table 5:** Results of follow-up interviews who RPh referred and referral was needed

Item	n (%)
Follow-up with doctor (n = 33)	
Patient did not go/no plan to go to doctor	10 (30.3)
Patient went/plan to go to doctor	23 (69.7)
Willingness to pay for service (n = 39)	
Yes	22 (56.4)
No	17 (43.6)
Payment for service (n = 22)	
$21–25	1 (4.5)
$16–20	3 (13.6)
$11–15	1 (4.5)
$5–$10	17 (77.2)

Percentages based on the number of valid data available

According to the follow-up survey, only ten patients (21 %) who were appropriately referred by the pharmacist followed through to see the physician within the 60 days post study. Within that time frame, the 11 respondents discussed the memory screening results with their physician, resulting in physicians conducting further assessment, primarily additional testing. The lack of follow-up indicates a need for more structured communications between the pharmacists and the physician related to cognitive memory concerns and a need to perform follow-up evaluations more than 60 days after the referral was made to identify all patients who follow-up at their next appointment with the physician.

Seventy-four screened patients (46 %) completed the voluntary participant satisfaction survey. The CMSRP was highly regarded by the patients who participated and completed the satisfaction survey. Of the respondents, 73 (98.6 %) reported that they were either very satisfied or satisfied with the program; 72 patients (97.2 %) reported that they were either very satisfied or satisfied with the information they received about memory and memory loss; 73 (98.6 %) were satisfied with the answers provided by the pharmacist to any question or concerns; 73 (98.6 %) were satisfied with the assistance of the pharmacist in the screening program; and 73 (98.6 %) indicated that the study pharmacy should continue to offer a cognitive memory-screening program. The same percentage indicated that they would recommend this program to family members or friends.

## Discussion

The detection of AD is difficult and without objective markers, often making diagnosis and treatment delayed. In a survey conducted by the Alzheimer’s Foundation of America, AD patients experienced symptoms for roughly 2 years and saw more than one doctor before obtaining a diagnosis [29]. Pharmacists can help to close the “patient identification gap” through basic memory screening assessments conducted in community pharmacy practice. Early and accurate diagnosis is an important step to ensuring the right treatment, care and support is received. This screening program allows at-risk patients to obtain a thorough assessment of cognitive memory decline and facilitates a medical diagnosis from their physician.

The three assessments used in community pharmacies are simple, straightforward and require a total of <7 min to administer. Most community pharmacies across the United States have similar workflow processes, which makes this model replicable in a wide variety of US community pharmacy practices. Just as many other pharmacist-provided screening programs are structured, core service elements included identification of appropriate patients to participate in the health screening, initial assessment for risk factors and family history, delivery of the screening service, patient counseling on screening results, and referral to a physician or other health care professional for appropriate follow-up. National organizations such as the Alzheimer’s Association and the national family caregivers Association have also developed excellent resources that can be used by pharmacists to help educate their patients.

An important aspect for providing expanded pharmacist services in any disease state is the potential revenue model. As a highly accessible health care provider, pharmacists are uniquely positioned to provide care services and community referral resources, encourage individuals and caregivers to take advantage of these services, and customize pharmacy services for those with AD. The exceptionally positive feedback from patients found in Table 5 indicates that the pharmacist should be appropriately compensated for these services. To ensure future sustainability of a CMSRP, more investigation is needed that explores the economic costs of the program relative to income generated.

The APhA Foundation’s White Paper on Expanding the Role of Pharmacists in Caring for Individuals with AD concluded that increased pharmacist involvement in the care of individuals with AD could improve clinical outcomes and family caregiver quality of life [30]. With the expected increase in the number of individuals diagnosed with AD, the resources and services to care for and support this population will be even further taxed. Maximizing the difference pharmacists can make in the lives of those who suffer from AD should include the continued development of innovative approaches for pharmacist involvement in AD, such as engaging in community awareness and advocacy, teaching and mentoring student pharmacists and pharmacy residents, participating in local Alzheimer’s Associations, and publishing and presenting professional activities.

## Limitations

Of the 161 patients, 16 (9.9 %) did not have referral status recorded on their assessment sheet. The pharmacist did not refer 19 (11.8 %) of patients whose tests indicated a need for referral due to the interaction with the patients or their refusal of a referral. Almost 79 % of patients referred to the physician did not follow-up within 60 days post-study. This indicates that a more effective physician communication strategy is needed to ensure appropriate physician follow-up for patients at-risk for AD. The response rate of 46 % to the patient satisfaction survey introduces potential bias to the survey results. Since the follow-up survey was voluntary, it is possible that those who were more satisfied with the service or those who visited the doctor as a result, provided feedback on the favorable experience. There is need for more research to examine if the CMSRP model might be applicable to other community settings both within and outside of the United States.

## Conclusion

The CMSRP was effective and valuable in identifying patients with cognitive memory decline who could be at-risk of developing AD and facilitating referral to their physicians. The high percentage of referred patients that did not follow-up with a physician underscores the importance of team-based patient care and open lines of communication between pharmacists and physicians. Additionally, patients were satisfied with the services provided by their pharmacist. Tools for the design and implementation of a cognitive memory screening and monitoring service in community pharmacies can be found at www.aphafoundation.org.
